# Comparative evaluation of anesthetic efficacy of 1.8 mL and 3.6 mL of articaine in irreversible pulpitis of the mandibular molar: A randomized clinical trial

**DOI:** 10.1371/journal.pone.0219536

**Published:** 2019-07-31

**Authors:** Stella Agra Silva, Anna Carolina Ratto Tempestini Horliana, Cláudio Mendes Pannuti, Paulo Henrique Braz-Silva, Carina Gisele Costa Bispo, Inês Aparecida Buscariolo, Rodney Garcia Rocha, Isabel Peixoto Tortamano

**Affiliations:** 1 Department of Stomatology, São Paulo University, São Paulo, SP, Brazil; 2 Postgraduate Program in Biophotonics Applied to Health Sciences, Nove de Julho University, São Paulo, SP, Brazil; 3 Department of Dentistry, Maringá State University, Maringá, Paraná, Brazil; University of Zurich, SWITZERLAND

## Abstract

**Objective:**

The aim of this study was to compare the anesthetic efficacy of two volumes of articaine in conventional inferior alveolar nerve block (IANB) of mandibular molars with irreversible pulpitis, and in cases of anesthetic failure, its complementation with periodontal ligament injection (PDL).

**Methods:**

Ninety patients with irreversible pulpitis in mandibular molars received conventional IANB with 1.8 mL or 3.6 mL of 4% articaine with 1:100,000 epinephrine. In cases of IANB failure, the same volumes were administered in the PDL. Presence of pulpal anesthesia and absence/presence of pain during pulpectomy were evaluated by electric pulp stimulation and verbal analogue scale, respectively. Relative risks (RR) with corresponding 95% confidence intervals (95% CI) were calculated for each outcome.

**Results:**

27% and 42% of the patients achieved pulpal anesthesia following IANB with 1.8 mL and 3.6 mL, respectively (RR = 0.63, CI 95% 0.35 to 1.14, p = 0.12). Analgesia during pulpectomy was present in 64% and 73% of the patients, respectively, for 1.8 mL and 3.6 mL (RR = 0.87, CI 95% 0.66 to 1.16, p = 0.36). In cases of IANB failure, 75% and 42% of the patients that received 1.8 mL and 3.6 mL of PDL injection, respectively, achieved pulpal anesthesia (RR = 1.80, CI 95% 0.87 to 3.72, p = 0.11). During a new attempt at the pulpectomy procedure, analgesia was present in 69% and 75% of the patients, respectively, for 1.8 mL and 3.6 mL (RR = 0.91, CI 95% 0.57 to 1.45, p = 0.71).

**Conclusion:**

Increasing the volume from 1.8 mL to 3.6 mL of the 4% articaine with 1:100,000 epinephrine in the IANB and in the PDL, did not significantly increase the success rate of pulpal anesthesia and clinical analgesia during the pulpectomy procedure. Therefore, both volumes presented a similar efficacy, though neither resulted in effective pain control during irreversible pulpitis treatment.

**Trial registration:**

ClinicalTrials.gov NCT02422823.

## Introduction

The conventional inferior alveolar nerve block (IANB) is the most commonly used technique for pulpal anesthesia in endodontic mandibular molar procedures [1–4]. However, IANB does not always result in successful pulpal anesthesia, and success is even lower when used for treatment of mandibular molars with irreversible pulpitis [3–8]. In certain situations when the failure occurs, it is necessary to use additional anesthetic techniques, such as intraosseous injection [7, 9–14], articaine mandibular buccal infiltration [14–17], intrapulpal injection [14], pre-emptive strategies to increase IANB success [14, 18, 19], and periodontal ligament injection [3, 11, 14, 20–22].

Some authors have suggested that the use of 2 anesthetic cartridges in the IANB (3.6 mL) with 2% lidocaine with different concentrations of epinephrine in asymptomatic teeth [23], as well as in teeth with irreversible pulpitis [24], results in more effective pulpal anesthesia than that performed with only 1 cartridge (1.8 mL). Other authors, when doubling the anesthetic volume, did not find a statistically significant difference in the success or failure of lidocaine use, regardless of the concentration of epinephrine used [25]. This was true for both asymptomatic [26] and symptomatic [2] teeth. Regarding comparisons of the doses used, we found only one study comparing different volumes (1.8 mL and 3.6 mL) of the 4% articaine solution with 1:100,000 epinephrine in the conventional IANB in mandibular molars with irreversible pulpitis [27].

Recently a systematic review concluded that increasing the volume of anesthetic agent from 1.8 to 3.6 mL significantly increased the success rate of IANB in mandibular molars with irreversible pulpitis [28]. However, only a very few number of research references were included in this review. Thus, we believe that more studies are necessary to clarify this subject.

The primary objective of this clinical trial was to compare the anesthetic efficacy of 1 cartridge (1.8 mL) and 2 cartridges (3.6 mL) of articaine 4% with epinephrine 1:100,000 in conventional IANB in patients with irreversible pulpitis in mandibular molars with spontaneous pain. The secondary objective was to compare the anesthetic efficacy of these different volumes in the periodontal ligament injection (PDL) as a complement in cases where the conventional IANB failed.

## Materials and methods

The study was approved by the University of São Paulo’s Dental School’s Committee for Ethics in Human Research (protocol # 626.279/2014). (S1 and S3 Files) and was registered on clinicaltrials.gov with the name of “Anesthetic Efficacy in Irreversible Pulpitis” (NCT02422823). Participants were recruited from São Paulo University’s dental clinics by a researcher who is not otherwise involved in the study. After written and verbal clarifications of the procedures, the voluntary nature of participation, and all risks and benefits of the study, patients signed a statement of informed consent that had been previously approved by the University of São Paulo Dental School’s Committee for Ethics in Human Research (https://osf.io/u2zx8/?view_only=6dcdcf8cf4fb46eb84c68811c2eadc22).

This is a randomized (1:1) controlled parallel single blind clinical trial. To ensure transparence and quality, the CONSORT (Consolidated Standards of Reporting Trials) recommendations were followed (S1 Checklist).

The sample size calculation was based on an 80% chance of detecting, with significance at the 5% level, a difference in success rate of 25% (65% control and 90% test). Ninety patients were recruited from April 2015 to June 2016, from the University of São Paulo’s Dental School’s Emergency Center. Recruitment was based on a diagnosis of irreversible pulpitis in the first or second mandibular molar participated with moderate to severe spontaneous pain, a positive response to the electric pulp test and a prolonged response to the thermal test with Endo-Frost (Coltene-Roeko, Langenau, Germany). Only healthy patients, according to the health history questionnaire, ranging in age from 18 to 50 years old, who had at least 1 adjacent tooth and a healthy contralateral canine tooth, and were without deep cavities, extensive restorations, advanced periodontal disease (insertion loss > 5 mm), and/or no history of trauma or sensitivity were included. Patients who used drug products that could interact with the local anesthetic were excluded from the study. No changes were made to the methods or trial outcomes after trial commencement.

**Fig 1:**
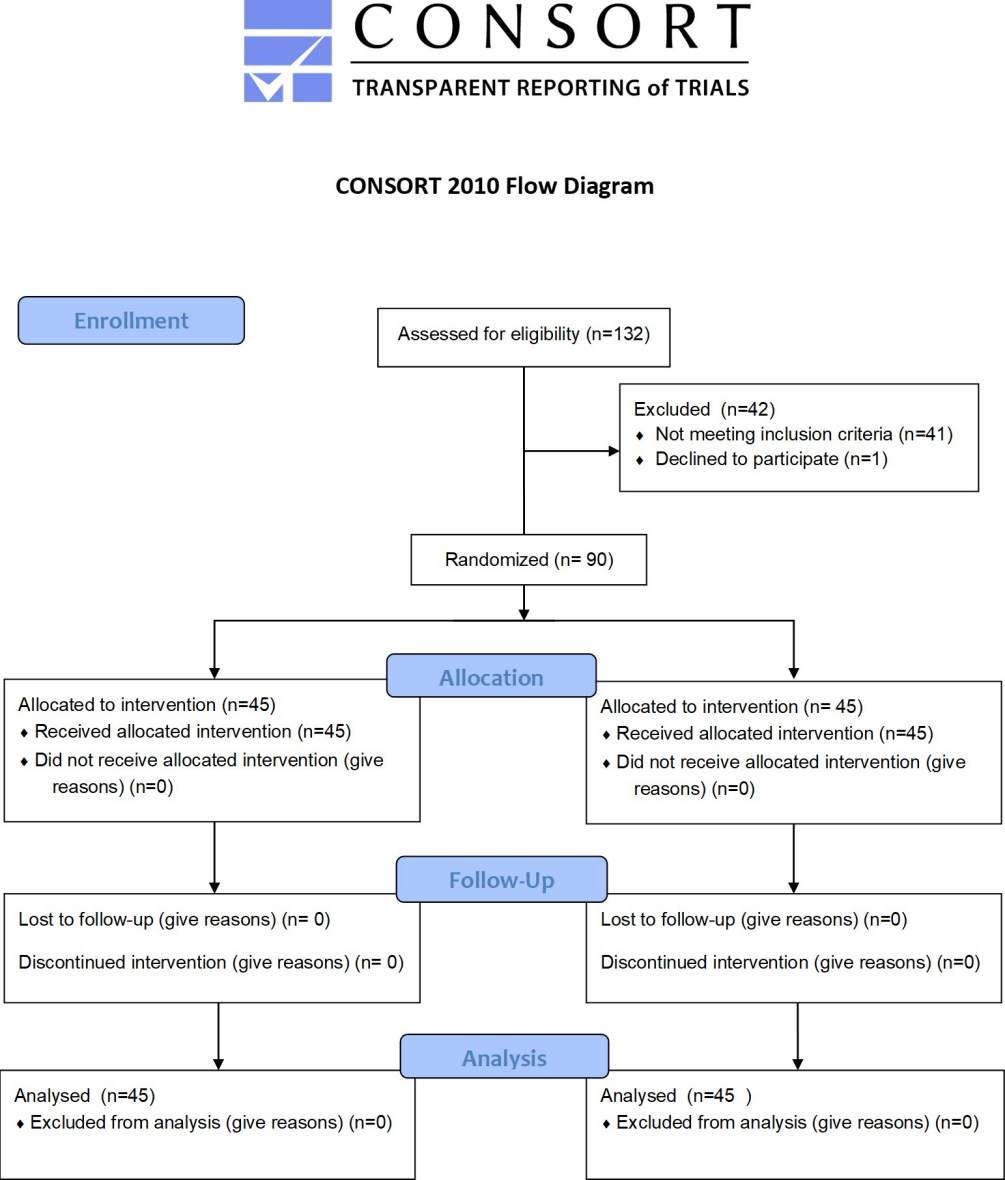
Activity flowchart.

The patients were randomized into 2 groups of 45 patients and received, at random (1:1), 1.8 mL or 3.6 mL of articaine hydrochloride 4% with epinephrine 1:100,000 (Articaine 100; DFL, Indústria e Comércio Ltda, RJ, Brazil) in the IANB (Fig 1).

We considered the 3.6 mL group the control group because at the University of São Paulo’s Dental School’s Emergency Center it is common to administer 2 cartridges in irreversible pulpitis. To ensure that the anesthetic volume was randomly chosen, we placed 45 brown envelopes in a box with 2 anesthetic tubes and 45 envelopes with 4 anesthetic cartridges. At the beginning of the anesthesia procedure, the operator (IPT) took, at random, a sealed brown envelope from the box. The electrical tests (SAS) and the pulpectomy procedure (IAB) were performed by different professionals, thus ensuring that they were not aware of the volume used and guaranteeing that the study was random and single blinded. Before anesthetic injection, the tooth with irreversible pulpitis, the adjacent tooth, and the contralateral canine had their pulp vitality tested twice with the electrical stimulator (Vitality Scanner 2006, SybronEndo, CA, USA). The contralateral canine was used as a parameter for analysis of the correct functioning of the equipment and to see if the patient was responding adequately. Two consecutive negative responses at maximal electrical stimulation (80uA) of the electrical pulp stimulator were the criteria that determined the success of pulpal anesthesia 10 minutes after IANB and immediately after the complementary periodontal ligament injection. Pulpal anesthesia, pulpectomy, and electrical tests were performed by three different professionals, throughout the study, each of them dedicated to a specific phase. For the conventional IANB injection, we used a side-loading carpule syringe and blood aspirator attached to a ring (Konnen; Kennen Indústria e comércio Ltda, SP, Brazil) as well as 27G disposable needles (Teruno Dental Needle; DFL, Indústria e comércio Ltda, RJ, Brazil). For the 3.6 mL group (2 cartridges), the second anesthesia was initiated immediately after the second step of the first anesthesia. Technique are detailed in supplemental material (protocol.io). Ten minutes after the IANB, the subjective signal of lip anesthesia was evaluated, by questioning the patient if his/her lip was numb. Immediately after questioning, a new electrical pulp vitality test was performed, and then preparation to access the cavity commenced. During the clinical procedure the patient was instructed to report any type of discomfort or pain, using a verbal analog scale where 0 = no pain; 1 = weak pain, mild and bearable; 2 = moderate and unbearable pain; 3 = severe, intense, and unbearable pain. The extent of access achieved when the patient felt pain was recorded as within the dentin, entering the pulp chamber, or during the pulpectomy procedure. Anesthesia was defined as successful when the professional could complete the pulpectomy without the patient reporting pain (pain scores 0 and 1). Pain scores of 2 and 3 were classified as failure of the IANB. This evaluation is the primary outcome measure. In these cases, we opted for PDL for anesthetic supplement.

In determining the failure of the IANB, we created a subgroup in which the PDL was necessary. Technique are detailed in supplemental material (protocol.io). This evaluation is a secondary outcome measure. The injections were administered by the same professional who had previously performed the IANB (IPT). Soon after completion of the anesthetic supplement, new electrical pulp vitality tests were performed. Subsequently, a new attempt at a pulpectomy was performed, in which the verbal analog scale was again used. Anesthesia success was defined as finishing the pulpectomy procedure without the patient reporting pain; in the case of a pain report, it was considered to be an anesthetic failure. At which point it was necessary to supplement with an intrapulpal injection. After 24 hours all patients were contacted by telephone by the urgent care center to register any adverse effects, related to paresthesia and postoperative pain. The distribution of sex, tooth type, and responses obtained with the electrical stimulator (negative or positive) and the pain scale after the IANB (no pain, 0 or 1, with pain, 2 or 3) of the 2 groups (1.8 mL e 3.6 mL) were compared using the χ^2^ test. Age distributions were analyzed using the Mann-Whitney test. The likelihood ratio test was applied to compare pain site distribution in the two groups after failure of the IANB as well as the responses obtained with the electrical stimulator (negative or positive) and the pain scale (analgesia) after the injection in the periodontal ligament. Relative risks (RR) with the corresponding 95% confidence intervals (95% CI) were calculated for each outcome. For all tests performed, the difference was assumed significant when P≤0.05.

## Results

All demographic data was presented in Table 1. The 1.8 mL group consisted of 60% female patients and the 3.6 mL group, 58%; age (the mean age for the 2 groups was 31 years old).

**Table 1:** Demographic data.

Demographic data	1.8 mL Group	3.6 mL Group	Total
Male / Female	18/27	19/26	37 / 53
Age (mean±SD)	30.8 ± 8.3	31.1± 8.2	30.9 ± 8.2
Tooth (1°/2° molar)	25/20	22/23	47 / 43

SD-standard deviation

All patients reported the subjective signal of lip anesthesia after 10 minutes of IANB. Immediately before the start of the pulpectomy procedure and after the application of the IANB, 12 patients from the 1.8 mL group (27%) and 19 patients from the 3.6 mL group (42%) achieved pulpal anesthesia (negative response to the maximum stimulation of 80 μA generated by the electrical stimulator). The use of 1.8 mL volume of anesthesia did not significantly increase the risk of achieving pulpal anesthesia (RR = 0.63, CI 95% 0.35 to 1.14, p = 0.12)

During the pulpectomy procedure, after the IANB, 29 patients from the 1.8 mL group (64%) and 33 from the 3.6 mL group (73%) didn’t report pain. The use of 1.8 mL volume of anesthesia was not significantly associated with analgesia during pulpectomy (RR = 0.87, CI 95% 0.66 to 1.16, p = 0.36)

Regarding the site of pain reported after the IANB, for the group receiving 1.8 mL pain was concentrated in the pulp chamber in 10 patients (62%), followed by pain in the dentin in 5 patients (31%), and pain in the root canal in 1 patient (6%) (Table 2). Similarly, in the 3.6 mL group, pain was also concentrated in the pulp chamber in 8 patients (67%), and the other 4 patients reported pain in the dentin (33%) (Table 2). Those values were not statistically significant (P = 0.6778).

**Table 2:** Frequency and percentage distribution of pain sites after doses of 1.8 mL and 3.6 mL after conventional IANB.

	Pain site			
Dose	Dentin	Pulp Chamber	Root Canal	Total
1.8 mL	5 (31%)	10(62%)	1 (6%)	16 (100%)
3.6 mL	4 (33%)	8 (67%)	0 (0%)	12 (100%)
Total	9 (32%)	18(64%)	1 (4%)	28 (100%)

% percentage

After PDL, 12 patients from the 1.8 mL group (75%) and 5 from the 3.6 mL group (42%) achieved pulpal anesthesia. The use of 1.8 mL volume of anesthesia increased the risk of achieving pulpal anesthesia, but this difference was not significant (RR = 1.80, CI 95% 0.87 to 3.72, p = 0.11).

During the new attempt at the pulpectomy procedure, 11 patients from the 1.8 mL group (69%) and 9 patients from the 3.6 mL group (75%) didn’t report pain, but these differences were not statistically significant (RR = 0.91, CI 95% 0.57 to 1.45, p = 0.71). All these results are presented in graphics in Suplemental material (S1 and S4 Tables).

The site of pain reported after the PDL in the 1.8 mL group was concentrated in the pulp chamber for 4 patients (80%) and the other patient reported pain in the dentin (20%). In the 3.6 mL group, we had an equal distribution among the 3 sites, and 1 patient experienced pain at each site (33% each), these differences were not statistically significant (P = 0.2456) and are shown in Table 3.

**Table 3:** Frequency and percentage of pain site distribution as reported for the 1.8 mL and 3.6 mL doses after complementary periodontal ligament injection.

	Pain site			
Dose	Dentin	Pulp chamber	Root Canal	Total
1.8 mL	1 (20%)	4 (80%)	0 (0%)	5 (100%)
3.6 mL	1 (33%)	1 (33%)	1 (33%)	3 (100%)
Total	2 (25%)	5 (62%)	1 (13%)	8 (100%)

% percentage

There were no important adverse events or unintended effects in either group of this study.

## Discussion

After 10 minutes of conventional IANB, profound lip anesthesia was verified in all patients (100%) for the 2 doses of articaine studied. However, pulpal anesthesia was not achieved in all patients at the 2 doses used. Therefore, lip anesthesia, often used as a clinical indicator of block success, is not a guaranteed signal of pulpal anesthesia; also, the onset of lip numbness might not indicate the onset of pulpal anesthesia [6, 16, 26, 29].

The maximal power use of the electric pulp stimulator (80μA) as criteria for pulpal anesthesia was based on previous studies [30, 31]. These studies showed that asymptomatic teeth and teeth with irreversible pulpitis present different results when tested. Negative responses to maximal pulp tester stimulation guaranteed pulpal anesthesia in asymptomatic teeth, similarly positive pulp tester responses resulted in pain during operative procedures. However, in symptomatic teeth, a negative response did not guarantee clinical analgesia during the pulpectomy procedure [30]. Other studies have also shown that pulpal anesthesia indicated by the electric pulp stimulator in teeth with irreversible pulpitis cannot be used as a reliable parameter for clinical analgesia [5–7, 10, 29]. This fact was confirmed in the present study, in which a patient from the 1.8 mL group and a patient from the 3.6 mL group presented a negative response to the pulp tester but felt pain during the pulpectomy procedure (therefore not undergoing clinical analgesia). However, the opposite also occurred; 18 patients from the 1.8 mL group and 15 patients from the 3.6 mL group presented a positive response to the pulp tester and, therefore, absence of pulpal anesthesia; however, they presented clinical analgesia during the pulpectomy procedure. Therefore, based on our clinical results and those reported in the literature [5–7, 10, 29] we can state that in the treatment of irreversible pulpitis of mandibular molars the electrical test is not a reliable indicator for the determination of pulpal anesthesia and analgesia. These false-positive responses, which may occur with the use of the electrical stimulator in irreversible pulpitis, still need to be investigated, insofar as they may involve various mechanisms of neuro-pulpal and neuroinflammatory interactions [32]. The pulp is formed by different types of nerve fibers, which present different responses to each stimulus. Non-myelinated type C fibers do not respond to electrical testing because of their high excitability threshold, so a stronger current is needed to stimulate them. However, the delta-A fibers are stimulated by the pulp tester because of their location, distribution, and higher conduction velocity [33].

Our study showed that neither of the two anesthetic volumes of articaine were sufficient to guarantee complete clinical analgesia during the pulpectomy procedure after the IANB. The results found in our study are in line with two studies that analyzed the anesthetic volume of 2% lidocaine in the IANB of patients with irreversible pulpitis [34]. In one of the studies [2], in which the authors reviewed 7 studies, they found that increasing the anesthetic volume from 1.8 mL to 3.6 mL did not lead to statistically significant differences in anesthetic success (28% and 39%). The other study [34] concluded that an increase in anesthetic volume of 2% lidocaine with epinephrine 1:80,000, increased anesthetic success after an IANB from 14.8% to 39.3%, but this result was not statistically significant.

Only two studies have found better results with increasing anesthetic volume in the IANB in patients with irreversible pulpitis and therefore are not in line with our results: Aggarwal *et al*. (2012) who evaluated the anesthetic volumes (1.8 mL and 3.6 mL) of lidocaine 2% with 1:200,000 epinephrine, and the study by Abazarpoor *et al*. (2015) which evaluated 4% articaine with 1:100,000 epinephrine. In the study by Abazarpoor *et al*. (2015) the authors found a statistically significant difference between the volumes of 1.8 mL and 3.6 mL, of 27.5% and 77.5%, respectively, for the success of pulpal anesthesia after the IANB with 4% articaine with epinephrine 1:100,000. It should be noted, however, that their study recruited patients with irreversible pulpitis, who did not present spontaneous pain, only prolonged response to the thermal test. This makes it difficult to compare to our study, because we recruited patients with irreversible pulpitis who reported spontaneous pain, and therefore probably presented a more exacerbated degree of inflammation.

It is very difficult to measure pain intensity. There is extensive literature regarding pain scales dating from the 1950s. Verbal Rating Scale (VRS), Visual Analogue Scale (VAS) or Numerical Rating Scale (NRS) are widely recommended for the assessment of pain intensity. However, VRS was preferred by the less educated patients [35,36]. They preferred the VRS, because they said it was easier than the VAS to understand and rate [36]. They also reported being more comfortable using words than numbers [36]. It is important to note that the patients studied here were from the University of São Paulo’s Dental School’s Committee for Ethics in Human Research. They are patients dependent on the Brazilian Public Healthcare Service for their care. According to some authors [37] Brazilian Single Healthcare System users, have only an elementary education (53.7%), belong to income class C (low-income) (50%), and depend on welfare (24.8%). Taking these points into consideration, we chose a verbal analog scale based on the Verbal Rating Scale (VRS), which has been cited in previous studies [5, 6, 29], to evaluate pain intensity in our study.

We found only two studies that indicated the site of the pain reported by the patient after the IANB [27,38]. In the study by Claffey *et al*. (2004) the pain site was predominantly in the dentin, 57% of articaine patients and 51% of lidocaine patients, followed by pain in the pulp chamber, 32% for articaine and 41% for lidocaine, and in the root canal, 11% for the articaine group and 7% for lidocaine group. The fact that pain occurred predominantly in the dentin led Claffey *et al*. (2004) to emphasize that clinicians would probably have difficulty administering supplemental techniques, such as intrapulpal anesthesia, in cases of failure of the IANB, requiring other techniques such as intraosseous and intraligamentary. However, in our study, after the IANB, a large number of the patients (62% and 67%, respectively, for the 1.8 mL and 3.6 mL groups) referred to pain only when the pulp chamber was accessed (Table 2).Thus we could have used intrapulpal injection, but we opted to supplement with the intraligamentary technique in order to evaluate its efficiency. In the study by Abazarpoor *et al*. (2015) the site at which the majority of patients reported pain during the endodontic procedure was in the pulp chamber, similar to our study. However, the difference between both volumes was significant, in the 1.8 mL group, 23 patients (57.5%) had pain in the pulp chamber while in the 3.6 mL group only 7 patients (17.5%) had pain, which differed from our study. In our study, there were no statistically significant differences between volumes and pain sites (Table 2).

A limitation of this study was not blinding the patients as to the number of injections used in this study. In the 3.6 mL group, two anesthetics cartridges were injected consecutively. In the 1.8 mL group only one anesthetics cartridge was injected. We could have injected 1.8mL of anesthetic + 1.8mL of saline in order to blind the experiment to the patient. However, we decide not to inject saline in order to simulate clinical conditions. Thus, we only blinded the researcher because the person that administered the anesthesia was not the same as the one who performed the clinical evaluations. Therefore this was a single blind study.

When analyzing complementary intraligamentary injections in cases of failure of the IANB in patients with irreversible pulpitis, we found only one study that used 1.7 mL of 4% articaine with 1:100,000 epinephrine without the use of a special syringe [21], and PDL injection achieved anesthetic success in 83.33% of the cases. In our study, clinical analgesia during the pulpectomy procedure, immediately after the injection of the periodontal ligament, was obtained in 69% of patients in the 1.8 mL group and 75% in the 3.6 mL group. However, these differences were not statistically significant, and the 8 patients who failed analgesia underwent intrapulpal anesthesia to complete the pulpectomy procedure, 5 from the 1.8 mL group and 3 from the 3.6 mL group. It should be noted that these results are to be generalized to young healthy adults, since they are the patients that normally present irreversible pulpitis.

The increase in anesthetic volume from 1.8 mL to 3.6 mL of articaine 4% with epinephrine 1:100,000 in the inferior alveolar nerve block (IANB) and in the supplemental anesthetic technique in the periodontal ligament (PDL) did not significantly increase the success rate of pulpal anesthesia and clinical analgesia during the pulpectomy procedure. However, it must be pointed out that the p-value was very close to 5% and therefore could be considered marginally significant. Future investigation related to the volume of the anesthetic solution of 4% articaine with 1:100,000 epinephrine, in the IANB and in the periodontal ligament injection, are still needed.

It is important to note that, in our study, a lower dose of the anesthetic solution was as effective as double the dose. Thus, clinicians should wait a few minutes before injecting the second cartridge allowing the local anesthetic time to propagate properly. Another option for a second injection may be a supplemental buccal infiltration injection of 4% articaine with 1: 100,000 epinephrine, according to the literature [14–17]. This could reduce the risk of paresthesia that can occur in the inferior alveolar nerve block.

Both volumes of 4% articaine with 1: 100,000 epinephrine (1.8 mL and 3.6 mL), in the IANB and in the PDL, presented similar efficacy, though neither were entirely efficient in controlling pain during treatment for irreversible pulpitis.

## Conclusions

We have concluded that an increase in anesthetic volume from 1.8 mL to 3.6 mL of articaine 4% with epinephrine 1:100 000 in the inferior alveolar nerve block (IANB) and in the supplemental anesthetic technique in the periodontal ligament (PDL) does not significantly increase the success rate for pulpal anesthesia and clinical analgesia during the pulpectomy procedure. Therefore both volumes presented a similar efficacy, though neither resulted in effective pain control for irreversible pulpitis treatment.

## Supporting information

S1 Checklist CONSORT statement. (PDF)

S1 File Project approved by the ethics committee in research. (DOC)

S2 File Original ethics committee in research (Portuguese). (PDF)

S3 File Original ethics committee in research (English). (PDF)

S1 Table A bar graph of responses to the pulp tester (percentage) in the 1.8 mL and 3.6 mL doses of 4% articaine hydrochloride with 1:100,000 epinephrine after the IANB. (TIF)

S2 Table A bar graph of the occurrence of pain (percentage) in the 1.8 mL and 3.6 mL doses of 4% articaine hydrochloride with 1:100,000 epinephrine after the IANB. (TIF)

S3 Table A bar graph of responses to the pulp tester (percentage) with 1.8 mL and 3.6 mL doses of 4% articaine hydrochloride with 1:100,000 epinephrine after periodontal ligament injection. (TIF)

S4 Table A bar graph of the occurrence of pain (percentage) with 1.8 mL and 3.6 mL doses of 4% articaine hydrochloride with 1:100,000 epinephrine after periodontal ligament injection. (TIF)

S1 Protocol Clinical trials protocol. (PDF)
