# Supplementary material for: Comparative evaluation of anesthetic efficacy of 1.8 mL and 3.6 mL of articaine in irreversible pulpitis of the mandibular molar: A randomized clinical trial
Source: PLoS One. 2019 Jul 31;14(7):e0219536. doi: 10.1371/journal.pone.0219536 (PMC6668778; doi:10.1371/journal.pone.0219536)
Supplement: S1 File — (DOC) [file pone.0219536.s002.doc]

**Title: Comparative evaluation of the anesthetic effectiveness of 1.8 mL and 3.6 mL of 4% articaine with 1:100.000 epinephrine on inferior alveolar nerve block in patients with irreversible pulpitis of mandibular molars**

**Advisor: Isabel de Freitas Peixoto**

**Student: Stella Agra da Silva**

**Abstract**

The objective of this study is to compare the anesthetic effectiveness between a 1.8 mL volume of 4% articaine hydrochloride with 1:100.000 epinephrine with a 3.6 mL of the same local anesthetic on Inferior Alveolar Nerve Block (IANB) in 70 patients of the Urgency Sector of the School of Dentistry of the Universidade de São Paulo with irreversible pulpitis of mandibular molars during pulpectomy procedure. The subjective signal of lip anesthesia, the presence of dental pulpal anesthesia and the absence of pain during the pulpectomy procedure, respectively, will be evaluated by; inquiring the patient, through the electric pulp stimulator (Vitality 2006® Scanner, SybronEndo, CA, USA), and through an analogue verbal scale. The data will be analyzed, most likely, by the Chi-square test and analysis of variance.

**Introduction and Justification**

The inferior alveolar nerve block (IANB) is the most frequently used technique

to achieve dental pulpal anesthesia during endodontic procedures on mandibular molars (1–7). However, the IANB not always results in a successful dental pulpal anesthesia, and the success is even lower when used for the treatment of mandibular molars with irreversible pulpitis (2, 8–12).

Over the years, several local anesthetics emerged, but none outperformed lidocaine regarding the efficacy and safety. Lidocaine was introduced in Dentistry in 1948 and to this day it is considered the “gold standard”, i.e., the drug to which all new local anesthetics are compared (13).

Articaine is the most recent drug introduced on the market and is being extensively studied due to its distinct molecular characteristics which increases its fat solubility, there are speculations that articaine presents an early action faster than lidocaine (14). However, some failures are also reported, such as a greater risk of paresthesia (15).

Nevertheless, some authors showed that there is no difference between 2% lidocaine and 4% articaine, both associated with 1:100.000 epinephrine, on IANB in patients with irreversible pulpitis (5, 12, 16). Attempting to solve this failure on IANB, high doses of local anesthetics are used, mainly in the treatment of irreversible pulpitis of lower molars. The higher the anesthetic dose applied by the professional, the greater the potential risk of toxic reactions. Cardiovascular and central nervous systems (CNS) are the primary targets of the toxicity from local anesthetics. We highlight that depending on the dose applied and the systemic condition of the patient, accidental intravenous injection may lead the patient to unpleasant sensations, and even coma or death (17).

Some authors suggest that using two anesthetic cartridges (3.6 mL) of 2% lidocaine with 1:50.000, 1:80.000 and 1:100.000 epinephrine concentrations in asymptomatic teeth (18) and with a concentration of 1:200.000 epinephrine in patients with irreversible pulpitis (19) resulted in better dental pulpal anesthesia than using one cartridge (1.8 mL). Other authors, when doubling the volume, found no statistically significant differences in the success or failure when using 2% and 4% lidocaine with 1:100.00 epinephrine (1) and with 2% lidocaine with 1:50.000 epinephrine in asymptomatic teeth (20) and in symptomatic teeth using 2% lidocaine with 1:100.000 epinephrine (21).

We found no studies that compared different volumes (1.8 mL and

3.6 mL) of the 4% articaine with 1:100.000 epinephrine solution in mandibular molars with irreversible pulpitis. Therefore, the objective of this clinical study is to compare the anesthetic effectiveness of one anesthetic cartridge (1.8 mL) and two cartridges (3.6 mL) of 4% articaine with 1:100.000 epinephrine in conventional block of the inferior alveolar nerve in patients with irreversible pulpitis in mandibular molars.

**Hypothesis**

To verify the equivalent effectiveness of 1.8 mL and 3.6 mL doses of 4% articaine hydrochloride with 1:100.000 epinephrine on IANB in patients with irreversible pulpitis in mandibular molars.

**Objectives**

**Primary**

- To compare the anesthetic effectiveness between a 1.8 mL volume of 4% articaine hydrochloride with 1:100.000 epinephrine and a 3.6 mL volume of the same anesthetic on IANB of patients with irreversible pulpitis in mandibular molars.

**Secondary**

- To compare the anesthetic effectiveness between a 1.8 mL volume of 4% articaine hydrochloride with 1:100.000 epinephrine and a 3.6 mL volume of the same anesthetic on the periodontal ligament in patients with irreversible pulpitis in mandibular molars when the first technique for IANB is unsuccessful (failure).

**Material and Methods**

**Material**

Human Material

**Patients selection:**

We will select 70 patients with irreversible pulpitis from the Urgency Sector, they must have the following characteristics:

- Healthy according to the health history questionnaire (anamnesis – Annex A) and verbal confirmation;

- Normoactive, with systolic blood pressure under 140 mmHg and diastolic blood pressure under 90 mmHg;

- Heart rate between 70-/+ 20 rate /minute;

- Age from 18 to 50 years;

- Who need pulpectomy on at least one mandibular molar;

- Who present at least one healthy adjacent tooth and one healthy contralateral canine or without the presence of deep caries, extensive restorations, advanced periodontal disease and with no history of trauma or sensitivity.

**Will be excluded from the study**

- Patients with a history of sensitivity to local anesthetics;

- Pregnant women or who suspect pregnancy;

- Those who are taking medications that can interact with the local anesthetic, such as anxiolytics, antidepressants, antipsychotics, antihistamines, analgesics and anti-inflammatory agents;

- Patients with septic process near the site of injection;

- Those under orthodontic treatment;

- People with heart diseases, neurological disease, hyperthyroidism and diabetes.

**On the teeth that will receive the pulpectomy**

- Mandibular molar teeth diagnosed with irreversible pulpitis, i.e., with spontaneous, moderate to severe, pain, with positive response to the electric pulp test and a prolonged response to the cold test (Endo-Frost – Contene – Roeko®, Germany).

**On the teeth that will undergo the electric test**

- The mandibular molar teeth diagnosed with irreversible pulpitis, a healthy adjacent tooth and the healthy contralateral canine, or without presence of deep caries, extensive restorations, advanced periodontal disease and with no history of trauma or sensitivity. The teeth will be insulated with cotton rolls and dried with air jets, the conductive gel for the electrocardiogram and ultrasound will be applied on the of the middle of the crown to establish contact between the tooth and the active tip of the device.

**Permanent Material**

**Infrastructure**

- Dental offices located in the Urgency Sector of the School of Dentistry of the Universidade de São Paulo.

**Equipment to perform the electric tests on the teeth pulp**

- Electric pulp tester Vitality Scanner 2006®, SybronEndo, CA, USA

**Clinical instruments**

**Instruments for the clinical examination**

- Dental mirror, double-ended explorer with straight tip and clamp

**Instruments for local anesthesia**

- Cartridge syringe with suction device (Konnen®; Kennen Indústria e

Comércio Ltda, São Paulo, Brazil)

**Instruments to perform the pulpectomy**

- Rotational instruments with irrigation (high rotation speed), spherical diamond tip KG Sorensen® 1019 or 1016 HL, Kerr and/or Hedstroem endodontic reamer, spatula to insert the provisional restorative material, endodontic aspiration cannula, luer taper for irrigation and dental curettes.

**Consumables**

**For the pulpectomy procedure**

- Cotton, zinc oxide-eugenol cement, 0.5% sodium hypochlorite (Dakin’s solution), intracanal PRP medication (2% para-monochlorophenol, rinosoro-polyethylene glycol 400-qsp 98% – Fórmula e Ação®)

**For local anesthesia**

- Long 27-gauge needle and short 30-gauge needle (Teruno Dental Needle® - DFL, Indústria e comércio Ltda)

- 4% articaine hydrochloride associated with 1:100.000 epinephrine (Articaine 100; DFL, Indústria e comércio Ltda).

- Topical gel – Benzocaine® 200 mg/g-20% (DFL, Indústria e comércio Ltda)

**To establish the electronic conduction between the tooth and the pulp from the “pulptester” – digital pulp tester**

- Conductive gel for electrocardiogram and ultrasonography (MedSystem®)

**For pulp vitality test to diagnose irreversible pulpitis**

- Endo-Frost – 200 ml (-50 °C) (Contene-Roeko® – Germany)

**For radiographic examination when needed**

- Radiographic film (periapical), Ektaspeed type (Kodak®)

**Methods**

The sample will be divided into two groups and two subgroups according to the volume of local anesthetic and to the anesthetic technique used. The existence of the subgroups depends on the failure of the first group, i.e., the first anesthetic technique used:

**Group A**

**A1** - Injection for inferior alveolar nerve block with 4% articaine hydrochloride with 1:100.000 epinephrine (n = 35) – 1.8 mL volume

**Subgroup A**

**A2** - Periodontal ligament injection with 4% articaine hydrochloride with 1:100.000 epinephrine (n = 35) – 1.8 mL volume + mandibular block injection held previously.

**Group B**

**B1** - Injection for inferior alveolar nerve block with 4% articaine hydrochloride with 1:100.000 epinephrine (n = 35) – 3.6 mL volume – 2 cartridges

**Subgroup B**

**B2** - Periodontal ligament injection with 4% articaine hydrochloride with 1:100.000 epinephrine (n = 35) – 3.6 mL volume – 2 cartridges + mandibular block injection held previously.

**Studied Variables**

- Subjective sign of lip anesthesia, presence of pulpal anesthesia and lack of pain after the IANB during the pulpectomy procedures;

- Presence of pulpal anesthesia and absence of pain after the complementary infiltration in the periodontal ligament, in cases of IANB failure.

**Obtaining and Registration of the Pulp Vitality Tests**

Pulp vitality tests will be performed on the tooth with irreversible pulpitis, on the adjacent tooth and on the contralateral tooth through the electric pulp stimulator (Vitality Scanner 2006®, SybronEndo, CA, USA). The pulp vitality monitor will turn on automatically as soon as contact between the tooth and the tip of the probe is established. To facilitate the electronic conduction, we will apply conductive gel used in electrocardiography and ultrasonography (MedSystem®) between the middle part of the tooth and the tip of the probe. The stimulus is perceived through a warm or tingling pulsatile pressure. The stream speed will be established in 25 seconds to increase from null power (0) until the maximum power (80). The indicator light on the probe emits an alert when maximum power is reached. The criterion for a successful pulpal anesthesia will be the negative response to the maximum stimulus of the pulp (80) for two consecutive times. The tests will always be performed two consecutive times in the following periods and (Annex B):

- immediately before the IANB anesthesia to obtain the mean base values of each tooth.

- ten minutes after the IANB, the patient will be asked about the lip numbness on this moment and immediately before the pulpectomy procedure.

- immediately after performing the additional anesthesia in the periodontal ligament, if necessary.

**Obtaining and Registration of the Subjective Lip Anesthesia**

The start and the duration of the lip anesthesia will be monitored through the question; “Is your lip numb?” from the end of the two consecutive injections of both cartridges (3.6 mL volume – Group B) or the injection of one cartridge (1.8 mL volume – Group A) to achieve IANB until the end of the 10-minute period, the pulpectomy procedures will start in this moment.

**Obtaining and Registration of Pain During the Pulpectomy Procedure**

The pain will be assessed using a verbal numerical scale from 0 to 3, recording the reported pain intensity and the proportion of access achieved (dentine, pulp chamber or on the canal) when the patient feel pain.

Numerical scale for pain assessment:

| **0** | **1** | **2** | **3** |
| --- | --- | --- | --- |

Absence Low pain Moderately Severe/Intense

of pain not uncomfortable uncomfortable unbearable

**Dynamics of the Experience**

Patients that seek the Urgency Sector of the School of Dentistry of the Universidade de São Paulo due to tooth pain will undergo a detailed anamnesis on their medical and dental history (Annex A).

After concluding the clinical diagnosis of irreversible pulpitis, i.e., the presence of spontaneous, moderate to severe pain and a prolonged response to the cold test (Endo-Frost®) in one lower molar of the mandible, which has a healthy adjacent tooth and a healthy contralateral canine or without the presence of deep caries, advanced restorations, periodontal disease and no history of trauma or sensitivity, the patient will be questioned on his/her spontaneous participation and collaboration in this clinical research. The informed consent form will be signed by the patient, confirming that his/her participation is voluntary (Annex C).

The pulp vitality tests will be performed two consecutive times on the tooth with irreversible pulpitis, on the adjacent tooth and on the contralateral canine before the administration of the anesthesia to obtain the mean base values (Annex B).

After applying the local topic anesthetic for 60 seconds on the retromolar region, one local anesthetic cartridge will be injected (1.8 mL) for Group A and two cartridges for Group B, for the IANB. The average time of injection will be approximately two (02) minutes for each cartridge.

Ten minutes after the injection, the patients will be asked about the deep lip numbness. After reporting deep lip numbness, the electric pulp vitality tests will be performed two consecutive times on the three teeth that were already mentioned. The non-anesthetized contralateral canine will be used as control to ensure that the “pulptester” is operating correctly and that the patients are responding appropriately. Two negative responses from the patients to the maximum power of the “pulptester” (80) will be used as the criterion for pulpal anesthesia (Annex B).

The research procedures will continue with the maneuvers to access the pulp chamber.

The patients will be instructed to report any discomfort during the opening procedure of the teeth, in this case the treatment will be interrupted immediately. The discomfort intensity will be reported using a numerical scale from 0 to 3, where zero is no pain, 1 weak or soft pain (pain is felt, but not uncomfortable), 2 moderate pain (uncomfortable, but not unbearable) and 3 severe (hard to tolerate, unbearable). The proportion of access achieved when the patient feel pain will be recorded as within the dentine, pulp chamber, or channel (Annex B).

Additional injections in the periodontal ligament (PDL) will be given to patients who feel pain. The injections will be mesial (mesial vestibular angle) and distal (mesial distal angle), and on the furcation region of the gingival sulcus through vestibular and lingual injections (mesial lingual angle, distal lingual angle and on the furcation region) of the tooth with irreversible pulpitis. The total amount of solution injected into the Group A will be approximately 0.9 mL for the vestibular surface and 0.9 mL for the lingual surface (approximately 0.3 mL for each injection). For Group B, the total amount of solution injected will be approximately 1.8 mL for the vestibular surface and 1.8 mL for the lingual surface (approximately 0.6 mL for each injection). The needle will be inserted in the gingival sulcus between the tooth and the bone crest at a 30-degree angle in relation to the long tooth axis and with the bevel facing the alveolar bone. The needle will be inserted with firm pressure until stopping, then, the anesthetic solution will be applied by pressure.

Immediately after the completion of the anesthesia in the periodontal ligament the electric pulp vitality tests will be performed again. If the pain persists after the additional injections, we will use an intrapulpal injection to allow the pulpectomy to be concluded.

The success of IANB will be defined as the ability to access the pulp chamber and perform the pulpectomy without reports of pain from the patient (0 and 1 on the scale) and failure will occur if the patient feels pain (2 and 3 on the scale). The success of the complementary anesthesia of the periodontal ligament will occur if the pulpectomy is completed without pain (0 and 1 on the scale) and failure if the patient reports pain (2 and 3 on the scale).

**Research stages**

**Diagnosis stage**

This stage will comprise the anamnesis, the clinical examination, the signature of the informed consent form and the definition of the base values of the electric pulp vitality tests for all three teeth (tooth with irreversible pulpitis, an adjacent tooth and the contralateral canine). The Numerical Pain Scale from 0 to 3 will be presented to the patients so they can familiarize themselves with it.

**Anesthetic stage 1**

This stage will comprise the topical application of the local anesthetic (20% Benzocaine), the injection of one cartridge (Group A) or consecutive injection of two cartridges (Group B) of IANB, the ten minutes after the injection to verify lip numbness, the electric pulp vitality tests to verify pulpal anesthesia and the pulpectomy procedure. If the patient reports any painful discomfort greater than 1 during the pulpectomy procedure, the proportion of access achieved will be recorded and the pulpectomy procedure will be interrupted to start the:

**Anesthetic stage 2**

This stage will start with injections on the periodontal ligament, they will be administered on the mesial vestibular, distal vestibular, mesial lingual, distal lingual and on the furcation region through vestibular and lingual injections. The total volume administered will be 1.8 mL on the vestibular and lingual surfaces for Group A and 3.6 mL volume for Group B. Electric pulp vitality tests will be performed again after the injections on the periodontal ligament and pulpectomy will be completed. If the periodontal ligament injection is not sufficiently effective for the pulpectomy procedure to be completed (2 and 3 on the scale), intrapulpal anesthesia will be used.

**Anesthetic Techniques**

**Standard technique of inferior alveolar nerve block (AINB)**

The technique will be based on the standard indirect technique for premolars and lower molars suggested by Malamed (2005), using a cartridge syringe with suction device and disposable 27-gauge needles. Blood aspiration tests will be performed at the start of each anesthesia procedure, as well as changing the needle position. After the initial 3-5 mm deep penetration, the blood will be aspirated and approximately 0.3 mL of the anesthetic solution will be injected. Then, the needle will be withdrawn a few millimeters and the cartridge will be carried to the opposite position in the region of the pre-molars. The needle is advanced toward the target location (close to the mandibular foramen) and when it touches the bone, the needle is withdrawn 1 mm. Aspiration is performed again and the remainder of the anesthetic solution will be injected, approximately 1.5 mL. The total injection time of the local anesthetic cartridge should be, approximately, two (02) minutes. For Group B, the second anesthesia (injection of one more cartridge-1.8 mL) will begin immediately after the end of the first.

**Periodontal ligament technique (complementary)**

For this technique, a normal cartridge syringe and disposable 30-gauge needles will be used. The injections will be administered on the vestibular surface of the gingival sulcus; mesial vestibular angle, mesial distal angle and furcation region; and on the lingual surface of the gingival sulcus; mesial lingual angle, distal lingual angle and on the furcation region of the tooth with pulpitis. The total amount of solution injected into the Group A will be 0.9 mL for the vestibular surface and 0.9 mL for the lingual surface (approximately 0.3 mL for each injection). The total amount of solution injected into the Group B will be 1.8 mL for the vestibular surface and 1.8 mL for the lingual surface (approximately 0.6 mL for each injection). The needle will be inserted in the gingival sulcus between the tooth and the bone crest at a 30-degree angle in relation to the long tooth axis and with the bevel facing the alveolar bone. The needle will be inserted with firm pressure until stopping, then, the anesthetic solution will be applied by pressure.

**Technique to Perform Pulpectomy Used by the Urgency Sector of the FOUSP**

The furcation of the crown must be performed using high-rotation instruments and long spherical diamond drills of the brand KG Sorensen, model 1019 HL. After accessing the pulp chamber, irrigation and aspiration using 0.5% sodium hypochlorite solution (Dakin’s solution) will be performed. What is left of the pulp chamber ceiling must be removed after the active end of the drill penetrates the pulp horns. The removal of the crown pulp must be done using dental curettes or the drill, then, another irrigation-aspiration process must be performed. The channels must be located and Kerr endodontic reamers (#8 to 20), preferably with 21 mm in length, depending on the diameter and length of the channels, will be introduced on the entrance of the channels. The penetration towards the apex must be slow and gradual, always considering the average length of the teeth and being far from it. The pulp removal will be done performing clockwise moves and only the number of turns required to the fold the pulp tissue, which usually is accomplished with one or two complete turns. The reamer must be removed from the channel and bring the pulp or its fragments. The mesial channels of the lower molars must be handled carefully, since when moving the reamer, they may be fractured, thus, the pressure from the reamer on the channel walls may bring pulp or fragments when being removed from the channel. Reamers of larger gauge or even a modified Hedstroem reamer may be need for the distal channel of the lower molars. However, since the full instrumentation of the channels will not be performed at that time, it is recommended to not exaggerate in instrumentation to create steps, hindering the further treatment of the respective tooth. Therefore, the purpose of the urgency treatment is only to perform the pulpectomy and provide pain relief without causing iatrogenesis.

**Form of Results analysis – Statistical Analysis**

Statistical analysis will be performed using the applications Minitab (version 16) and SPSS (version 18). The data will be analyzed, most likely, by the Chi-square test and analysis of variance. All statistical methodology used can be found in Fisher and van Belle (1993).

**Work Plan and Schedule of execution**

The project is structured to be held in 9 months, as shown in the following work plan.

| **Activities** | **2014 Months** | | | | | | | | | | | |
| --- | --- | --- | --- | --- | --- | --- | --- | --- | --- | --- | --- | --- |
|  | **1** | **2** | **3** | **4** | **5** | **6** | **7** | **8** | **9** | **10** | **11** | **12** |
| Submission for assessment by the CEP |  |  |  | X | X |  |  |  |  |  |  |  |
| Clinical care of the patients |  |  |  |  |  | X | X | X | X |  |  |  |
| Tabulation of Results |  |  |  |  |  |  |  |  |  | X |  |  |
| Statistical Analysis |  |  |  |  |  |  |  |  |  |  | X |  |
| Drafting the article and conclusion |  |  |  |  |  |  |  |  |  |  |  | X |

**Risks**

Given that several questions about the medical history of patients will be made, mainly regarding their blood pressure, cardiovascular disease, allergies and medications used, it will be very difficult for any complications derived from the anesthesia to occur because the use of these drugs is very safe, especially in healthy individuals and in the doses used.

Discomfort (slight tingle sensation, a very light shock) resulting from the application of the electric test may occur. Pain caused by the needle puncture and the injection of the local anesthetic may also occur and should be minimized by the application of the topical anesthetic gel before the puncture.

**Benefits**

If we find that there is no difference between the use of 1 or 2 cartridges, we will be able to, in the future, safely inject a lower amount of local anesthetic in the IANB of mandibular molars with irreversible pulpitis.

**Primary outcome**

The presence of analgesia after the IANB is similar between the doses used and does not achieve 100% success rate

**Secondary outcome**

The presence of pulpal anesthesia after the IANB is similar between the doses used and does not achieve 100% success rate

**References**

1. Vreeland DL, reader A, Beck M, et al. An evaluation of volumes and concentrations of lidocaine in human inferior alveolar nerve blocks. J Endod 1989; 15:6-12.

2. Cohen HP, Cha BY, Spanberg LS. Endodontic anesthesia in mandibular molars: a clinical study. J Endod 1993;19:370-3.

3. Childers M, Reader A, Nist R, et al. Anesthetic efficacy of the periodontal ligament injection after an inferior alveolar nerve block. J Endod 1996;22:317-20.

4. Dagher BF, Yared GM, Machtou P. The anesthetic efficacy of volumes of lidocaine in inferior alveolar nerve blocks. J Endod 1997;23:178-80.

5. Claffey E, Reader A, Nusstein J, Beck M, Weaver J. Anesthetic efficacy of articaine for inferior alveolar nerve blocks in patients with irreversible pulpitis. J Endod 2004;30:568-71.

6. Mikesell P, Nusstein J, Reader A, et al. A comparison of articaine and lidocaine for inferior alveolar nerve blocks. J Endod 2005;31:265-70.

7. Goldberg S, Reader A, Drum M, et al. Comparison of the anesthetic efficacy of the conventional inferior alveolar, Gow-Gates, and Vazirani-Akinosi techniques. J Endod 2008;34:1306-11.

8. Aggarwal V, Jain A, Debipada K. Anesthetic efficacy of supplemental buccal and lingual inflitrations of articaine and lidocaine after an inferior alveolar nerve block in patients with irreversible pulpitis. J Endod 2009;35:925-29.

9. Reisman D, Reader A, Nist R, Beck M, Weaver J. Anesthetic efficacy of the supplemental intraosseus injection of 3% mepivacaine in irreversible pulpitis. Oral Surg Oral Med Oral Pathol 1997;84:676-82.

10. Nusstein J, Reader A, Nist R, Beck M, Meyers WJ. Anesthetic efficacy of the supplemental intraosseous injection of 2% lidocaine with 1:100,000 epinephrine in irreversible pulpitis. J Endod 1998;24:487-91.

11. Kennedy S, Reader A, Nusstein J, Beck M, Weaver J. The significance of needle deflection in sucess of the inferior alveolar nerve block in patients with irreversible pulpits. J Endod 2003;29:630-3.

12.Tortamano IP, Siviero M, Costa CG, Buscariolo IA, Armonia PL. A comparison of the anesthetic efficacy of articaine and lidocaine in patients with irreversible pulpitis. J Endod 2009;35:165-8.

13. Fuzier R, Lapeyre-Mestre M, Samii K, Montastruc JL. Adverse drug reactions to local anaesthetics: a review of the French pharmacovigilance database. Association of Regional Pharmacovigilance Centres Drug Saf. 2009;32(4):345-56.

14. Malamed SF, Gagnon S, Leblanc D. Articaine hydrochloride: a study of the safety of a new amide local anesthetic. J Am Dent Assoc 2001; 132(2): 177-185.

15. Hass DA, Lennon D. Local anesthetic use by dentists in Ontario. J Can Dent Assoc 1995; 61(4): 297-304.

16. Sherman MG, Flax M, Namerow K, Murray PE. Anesthetic efficacy of the gow-gates injection and maxillary infiltration with articaine and lidocaine for irreversible pulpitis. J Endod 2008;34:656-9.

17. Malamed SF.Ações clínicas de agentes específicos. In: Malamed SF. Manual de anestesia local.Rio de Janeiro: Elsevier; 2005.p.55-82.

18. Yared GM, Dagher FB.Evaluation of lidocaine in human inferior alveolar nerve block J Endod. 1997 Sep;23(9):575-8.

19. Aggarwal V, Singla M, Miglani S, Kohli S, Singh S. Comparative evaluation of 1.8mL and 3.6mL of lidocaine with 1:200,000 epinephrine for inferior alveolar nerve block in patients with irreversible pulpitis: a prospective, randomized sinle-blind study. J Endod. 2012 Jun;38(6):753-6.

20. Wali M, Drum M, Reader A, Nusstein J. Prospective, randomized single-blind study of the anesthetic efficacy of 1.8 and 3.6 mililiters of 2% lidocaine with 1:50,000 epinephrine for inferior alveolar nerve block J Endod. 2010 Sep;36(9):1459-62.

21. Fowler S, Reader A. Is a volume of 3.6mL better than 1.8mL for inferior alveolar nerve blocks in patients with symptomatic irreversible pulpitis? J Endod. 2013 Aug;39(8):970-2.

22. Fisher, L, van Belle, G. 1993. Biostatistics. John Wiley & Sons, New York.

**Annex A: Anamnesis file**

Name_____________________________________________________ ID

Address

Zip Code________________ District___________________Phone:

Birth date___/___/____ Marital status__________Profession_______________Color

Medical History: Date__/___/___

1) Are you feeling some kind of pain or discomfort at the moment?

2) Are you undergoing some form of medical treatment these days?______Why?

3) Are you taking any medication? Which one(s)?

4) Have you ever had an allergic reaction to any drug, food, sulfur or other product?

5) Have you ever undergone local anesthesia?

6) Did you have some type of adverse reaction to this anesthesia?

7) Have you ever undergone a surgery or been hospitalized?

8) Have you ever received blood transfusion?

9) Have you ever suffered a hemorrhage?

10) Which of these symptoms or illnesses do you have or have ever had?

Hypertension Pneumonia Diabetes

Hypotension Tuberculosis Weight loss (+5 kg)

Headache Sinusitis Weight gain (+5 kg)

Asthma Rheumatic fever Hepatitis or jaundice

Bronchitis Gastritis Liver disorders

Epilepsy Anemias Sexually transmitted diseases

Kidney problems Fainting Psychic disorders

11) Do you feel tired often?_______Shortness of breath?

12) Do you have shortness of breath when lying down?

13) Do you feel chest pain after an effort or under stress?

14) What was the last time you measured your arterial pressure?______________How it was?

15) Do you have any congenital or acquired heart disease (Chagas disease, for example)?

16) Do you have or has had any symptoms or illnesses that were not mentioned above?

17) Are you pregnant?

18) Do you smoke?

19) Do you make use of alcohol or any other drugs?

20) Have you ingested alcohol in the last 4 hours?

For all legal purposes, I declare that I provided correct information about my state of health, I omitted nothing on the questionnaire I answered.

Sign._____________________________________ date ____/____/____

**Annex B: pulptester records**

Patient:_________________________________________ date __/__/__

Solution and dose (.....) Articaine 1.8 mL (.....) Articaine 3.6 mL

Technique (...) local block

(...) local block complemented with PDL injection

Treated tooth:_______________

**Diagnosis stage**

| Teeth | **VT1** | **VT2** | **M** |
| --- | --- | --- | --- |
| Canine |  |  |  |
| Adjacent tooth |  |  |  |
| Tooth-pulpectomy |  |  |  |

**VT1 and VT2 = Vitality test 1 and 2, M = mean**

In this phase we will present the verbal numerical pain scale to the patients

Numerical scale for pain assessment:

| **0** | **1** | **2** | **3** |
| --- | --- | --- | --- |

Absence Light pain Moderate Severe/Intense

of pain pain pain

**Anesthetic stage 1**

End of the anesthesia____:______h

Lip anesthesia ( ) Present ( ) Absent

Time:10:00 min / Hour:_____:_____h

Pulpal anesthesia

| Teeth | **VT1** | **VT2** | **M** |
| --- | --- | --- | --- |
| Canine |  |  |  |
| Adjacent tooth |  |  |  |
| Tooth-pulpectomy |  |  |  |

Pulpectomy

Mark an X in the column corresponding to the number that the patient attributes to pain

Verbal numerical pain scale:

| Local | **0** | **1** | **2** | **3** |
| --- | --- | --- | --- | --- |
| Dentine |  |  |  |  |
| Pulp chamber |  |  |  |  |
| Channel |  |  |  |  |

Success ( ) Failure ( )

**Anesthetic stage 2:**

Depends on the failure of the anesthetic stage 1.

Pulpal anesthesia

| Teeth | **VT1** | **VT2** | **M** |
| --- | --- | --- | --- |
| Canine |  |  |  |
| Adjacent tooth |  |  |  |
| Tooth-pulpectomy |  |  |  |

Pulpectomy

Mark an X in the column corresponding to the number that the patient attributes to pain

Verbal numerical pain scale:

| Local | **0** | **1** | **2** | **3** |
| --- | --- | --- | --- | --- |
| Dentine |  |  |  |  |
| Pulp chamber |  |  |  |  |
| Channel |  |  |  |  |

Success ( ) Failure ( )

**ANNEX C – Informed Consent Form**

**UNIVERSIDADE DE SÃO PAULO**

**SCHOOL OF DENTISTRY**

INFORMED CONSENT FORM

**Title:**

Comparative evaluation of the anesthetic effectiveness of 1.8 mL and 3.6 mL of articaine hydrochloride with 1:100.000 epinephrine on inferior alveolar nerve block in patients with irreversible pulpitis of mandibular molars

**Researchers:**

Isabel de Freitas Peixoto e Stella Agra da Silva

**Experiment Location:**

The study will be performed on the Urgency Sector of the School of Dentistry of the Universidade de São Paulo.

**Invitation for the voluntary participation in the study:**

You are being invited to participate, voluntarily, that is, if you want to, of this research, and if at any moment you wish to stop participating, you have total freedom to withdraw this consent, without any detriment to the continuity of the treatment.

**Objectives of the study:**

When treating lower molar teeth with inflammation in their nerve, which we call “irreversible pulpitis”, anesthesia is not always efficient. The most frequent anesthesia used for the treatment of lower molars with irreversible pulpitis is a local anesthesia in which we penetrate the needle behind the last lower molar erupted, this procedure is known as the Inferior Alveolar Nerve Block (IANB). There are studies that suggest the use of 2 anesthetic cartridges for a most effective anesthesia, but other studies report that there is no significant difference when the volume of these anesthetic solutions is doubled. Given that we did not find any study comparing the use of different volumes of the local anesthetic 4% articaine hydrochloride with 1:100.000 epinephrine (1.8 mL, represented by 1 cartridge and 3.6 mL represented by 2 cartridges) in mandibular molar with irreversible pulpitis, this study will be performed to investigate this fact. Therefore, the objective of this study will be:

- To compare the anesthetic effectiveness between a 1.8 mL (1 cartridge) volume of 4% articaine hydrochloride with 1:100.000 epinephrine and a 3.6 mL (2 cartridges) volume of the same anesthetic on IANB of patients with irreversible pulpitis in mandibular molars.

- To compare the anesthetic effectiveness between a 1.8 mL volume of 4% articaine hydrochloride with 1:100.000 epinephrine and a 3.6 mL volume of the same anesthetic on the periodontal ligament in patients with irreversible pulpitis in mandibular molars when the first technique for IANB is unsuccessful (failure).

**Procedures:**

We will select 70 volunteers with normal blood pressure and normal health, of both sexes, from 18 to 50 years of age. These patients must present a posterior lower tooth with inflammation of the nerve, a posterior tooth next to it (adjacent) and a canine tooth on the other side, which are free from caries, the presence of extensive restorations and gum disease adjacent to the tooth. We will perform electric tests on these teeth to assess if the effects of the local anesthetic are present. The tooth on which will be the pulpectomy procedure will be performed (removal of the inflamed nerve) is the tooth that is in pain. The pain relief procedures performed, if you accept to participate in the research, will be exactly those that are already done by the Urgency Sector of the FOUSP. The difference is that in addition to the thermal test with cold, which is usually done as a routine procedure during the clinical examination of tooth pain, we will also perform electric tests.

We will not accept patients who are pregnant, allergic to the substances used in the research, who have cardiac diseases and high blood pressure and who have taken (ingested) any kind of analgesic. The apparatus used for the electrical testing will be the Vitality Scanner 2006®, SybroEndo, CA, USA, also known as “pulp tester”, its tip (probe) will be placed on the crown of the tooth that is in pain, the adjacent tooth next and the canine on the other side. The anesthetic used will be:

ARTICAINE 100® (DFL) – 4% articaine hydrochloride associated with 1:100.000 epinephrine

**Expected risks and discomforts:**

Discomfort (slight tingle sensation, a very light shock) resulting from the application of the electric test may occur. Pain caused by the needle puncture and the injection of the local anesthetic may also occur and should be minimized by the application of the topical anesthetic gel before the puncture. If you feel pain during the procedure, you must notify the researcher and the procedure will be interrupted.

**Time:**

The time required to perform this research will be virtually the same as the time we would take to perform the urgency service, which is the reason why you came to us for the resolution of your pain. The only difference will be an increase of approximately 6 minutes, during which we will perform the electrical testing applications in the respective teeth.

**Benefits of the experiment:**

This research will allow us to assess whether there is a significant difference in the injected quantity of anesthetic; 1.8 mL (1 cartridge) or 3.6 mL (2 cartridges), during the pulpectomy procedure (removal of the nerve) that provides a greater clinical efficacy of the anesthetic. If we find that there is no difference between the use of 1 or 2 cartridges, we will be able to, in the future, safely inject a lower quantity of local anesthetic. Other direct benefit to the patient will be the pulpectomy procedure for pain relief, which will be done regardless of the patient’s participation in the research or not.

**Financial aid:**

We will not provide any kind of financial aid since you came to us voluntarily for the relief of your pain, and there will be no return to conclude this research.

**Confidentiality guarantee:**

Any and all information obtained in during the research will be confidential to the responsible researchers.

**Right to abandon:**

You have the freedom to withdraw your consent and no longer participate in the study at any moment, and you will still be able to receive the benefits from the research, that is, receive the emergency care.

**Contact:**

The voluntary patient is guaranteed to receive responses to any questions or clarification of the procedures. Given that several questions about the medical history of patients will be made, mainly regarding their blood pressure, cardiovascular disease, allergies and medications used, it will be very difficult for any complications derived from the anesthesia to occur because the use of these drugs is very safe, especially in healthy individuals and in the doses used. However, we are able to provide first aid if any medical emergency occurs, and eventually, we can also count on a nurse and on the Emergency Room of the University Hospital. After the urgency care, the patients will be forwarded to complete the endodontic treatment on the Dental Clinic of the FOUSP, given the availability of vacancy and, if there is a need for a new urgency intervention (postoperative pain or discomfort), the researcher and the emergency professors will be available (within the consultation hours of the sector – from 8 AM to 8 PM).

We will also available for any information or complaints you have on the following phone numbers: +55 (11) 3091-8030 or +55 (11) 3091-7813

If you have any questions regarding the ethics of this research, you may contact the Research Ethics Committee of the School of Dentistry:

Av. Lineu Prestes 2227, 05508-000 São Paulo, telephone +55 (11) 3091-7960, e-mail cepfo@usp.br

**Written Informed Consent:**

I_____________________________________, ID___________________, certify that, having read the information and being sufficiently clarified on the items of the research: “Comparative evaluation of the anesthetic effectiveness of 1.8 mL and 3.6 mL of articaine hydrochloride with 1:100.000 epinephrine on inferior alveolar nerve block in patients with irreversible pulpitis of mandibular molars” under the responsibility of the researcher Stella Agra da Silva and under the guidance of Ph.D. Professor Isabel de Freitas Peixoto, I fully agree with the performance of the experiment. Therefore, I authorize the execution of the research work described above with my spontaneous collaboration.

_____________________ _________________________

Patient’s signature Researcher’s signature

(Duplicate – Researcher)

**Written Informed Consent:**

I_____________________________________, ID___________________, certify that, having read the information and being sufficiently clarified on the items of the research: “Comparative evaluation of the anesthetic effectiveness of 1.8 mL and 3.6 mL of articaine hydrochloride with 1:100.000 epinephrine on inferior alveolar nerve block in patients with irreversible pulpitis of mandibular molars” under the responsibility of the researcher Stella Agra da Silva and under the guidance of Ph.D. Professor Isabel de Freitas Peixoto, I fully agree with the performance of the experiment. Therefore, I authorize the execution of the research work described above with my spontaneous collaboration.

_____________________ _________________________

Patient’s signature Researcher’s signature
