# Supplementary material for: Comparative evaluation of anesthetic efficacy of 1.8 mL and 3.6 mL of articaine in irreversible pulpitis of the mandibular molar: A randomized clinical trial
Source: PLoS One. 2019 Jul 31;14(7):e0219536. doi: 10.1371/journal.pone.0219536 (PMC6668778; doi:10.1371/journal.pone.0219536)
Supplement: S2 File — (PDF) [file pone.0219536.s003.pdf]

PARECER CONSUBSTANCIADO DO CEP

DADOS DO PROJETO DE PESQUISA

Título da Pesquisa: Avaliação comparativa da eficácia anestésica da articaína com epinefrina no bloqueio do nervo alveolar inferior em pacientes com pulpite irreversível de molares mandibulares

Pesquisador: Isabel de Freitas Peixoto

Área Temática:

Versão:

CAAE: 30188014.7.0000.0075

Instituição Proponente: Universidade de Sao Paulo

Patrocinador Principal: Financiamento Próprio

DADOS DO PARECER

Número do Parecer: 626.279

Data da Relatoria: 25/04/2014

Apresentação do Projeto:

O protocolo de anestesia por Bloqueio do Nervo Alveolar Inferior (BNAI) recomenda o uso de dois (02) tubetes anestésicos, mas segundo a pesquisadora não há descrito na literatura fundamentação cientificística segura para tal rotina. Dessa forma o estudo clínico propõem avaliar a eficácia anestésica com dois (02) volumes de medicamento.

Objetivo da Pesquisa:

Primário

- Comparação da eficácia anestésica de um volume de 1,8mL do cloridrato de articaína 4% com epinefrina 1:100.000 com um volume de 3,6mL do mesmo anestésico no BNAI em pacientes com pulpite irreversível de molares mandi-bulares.

Secundário

- Comparação da eficácia anestésica de um volume de 1,8 mL do cloridrato de articaína 4% com epinefrina 1:100.000 com um volume de 3,6mL do mesmo anestésico na injeção do ligamento periodontal em pacientes com pulpite irre-versível de molares mandibulares quando a primeira técnica para o BNAI tiver insucesso (falhar).

Endereço: Av Prof Lineu Prestes 2227
Bairro: Cidade Universitária **CEP:** 05.508-900
UF: SP **Município:** SAO PAULO
Telefone: (11)3091-7960 **Fax:** (11)3091-7814 **E-mail:** cepfo@usp.br

Avaliação dos Riscos e Benefícios:

Riscos e benefícios mensurados e apresentados de forma clara e adequada tanto no projeto como no Termo de Consentimento Livre e Esclarecido (TCLE).

Comentários e Considerações sobre a Pesquisa:

Pesquisa tem o potencial de jogar luz a uma rotina clínica que pode estar superdimensionada para as necessidades de um serviço de urgência, e portanto, se, se comprovar a hipótese de eficacia com uso de apenas um (01) tubete anestésico os pacientes serão submetidos a menor quantidade de medicamento diminuindo eventuais riscos e/ou desconfortos com o tempo do efeito anestésico.

Considerações sobre os Termos de apresentação obrigatória:

TCLE, Folha de Rosto e Autorização do Supervisor da Clinica Odontológica da FOUSP. Adequados.

Recomendações:

Tendo em vista a legislação vigente, devem ser encaminhados ao CEP-FOUSP relatórios parciais semestrais referentes ao andamento da pesquisa e relatório final ao término do trabalho. Qualquer modificação do projeto original deve ser apresentada a este CEP, de forma objetiva e com justificativas, para nova apreciação.

Conclusões ou Pendências e Lista de Inadequações:

Projeto bem estruturado em suas hipóteses e metodologias.

Situação do Parecer:

Aprovado

Necessita Apreciação da CONEP:

Não

Considerações Finais a critério do CEP:

SAO PAULO, 25 de Abril de 2014

Assinador por:
Maria Gabriela Haye Biazevic
(Coordenador)

Endereço: Av Prof Lineu Prestes 2227
Bairro: Cidade Universitária **CEP:** 05.508-900
UF: SP **Município:** SAO PAULO
Telefone: (11)3091-7960 **Fax:** (11)3091-7814 **E-mail:** cepfo@usp.br
