# Supplementary material for: Comparative evaluation of anesthetic efficacy of 1.8 mL and 3.6 mL of articaine in irreversible pulpitis of the mandibular molar: A randomized clinical trial
Source: PLoS One. 2019 Jul 31;14(7):e0219536. doi: 10.1371/journal.pone.0219536 (PMC6668778; doi:10.1371/journal.pone.0219536)
Supplement: S3 File — (PDF) [file pone.0219536.s004.pdf]

UNIVERSIDADE DE SÃO PAULO
FACULDADE DE ODONTOLOGIA

Comitê de Ética em Pesquisa
Tel. (55.11) 3091 7960
cepfo@usp.br

UNIVERSITY OF SÃO PAULO (USP)
SCHOOL OF DENTISTRY (FO)
ETHICS COMMITTEE ON INVESTIGATION (CEP)

APPROVAL
CAAE 30188014.7.0000.0075 / 626.279

Based on the ***Ethics Committee on Investigation*** members dicussion, this Committee has ***APPROVED*** the investigation protocol **"Comparative evaluation of the anesthetic efficacy of articaine with epinephrine in inferior alveolar nerve block in patients with irreversible pulpitis of mandibular molar"** under responsability of Isabel de Freitas Peixoto (Isabel Peixoto Tortamano).

Considering the current legislation, this Committee must receive annual reports with regard the on going results. Any amendment on the original Project must be presented to this CEP with clear identification of the changes.

São Paulo, May 3, 2018.

Maria Gabriela Haye Biazevic, DDS, PhD
FOUSP CEP´s Director
