# Supplementary figures and images for: Comparative evaluation of anesthetic efficacy of 1.8 mL and 3.6 mL of articaine in irreversible pulpitis of the mandibular molar: A randomized clinical trial
Source: PLoS One. 2019 Jul 31;14(7):e0219536. doi: 10.1371/journal.pone.0219536 (PMC6668778; doi:10.1371/journal.pone.0219536)

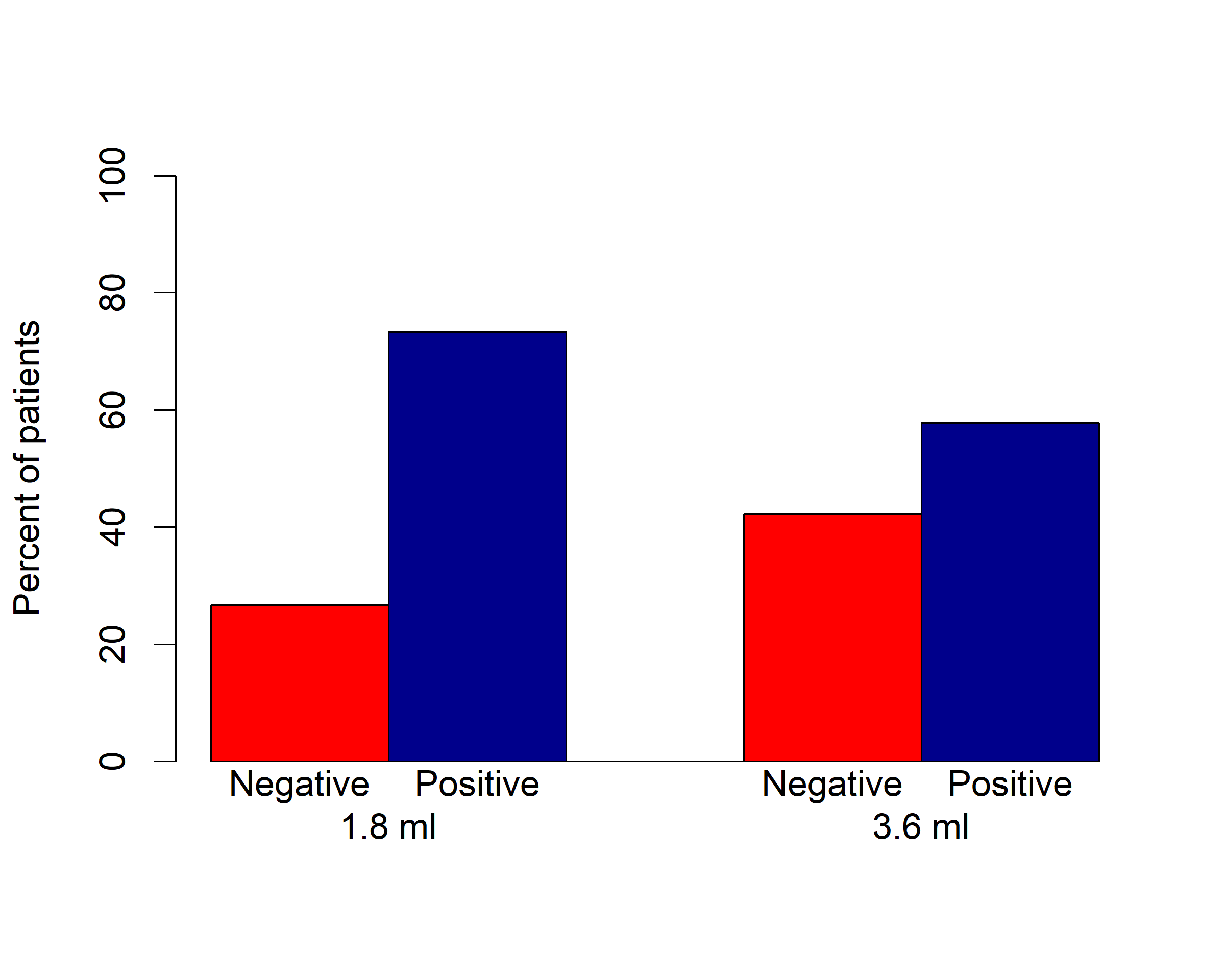

Supplement: S1 Table — (TIF) [file pone.0219536.s005.TIF]

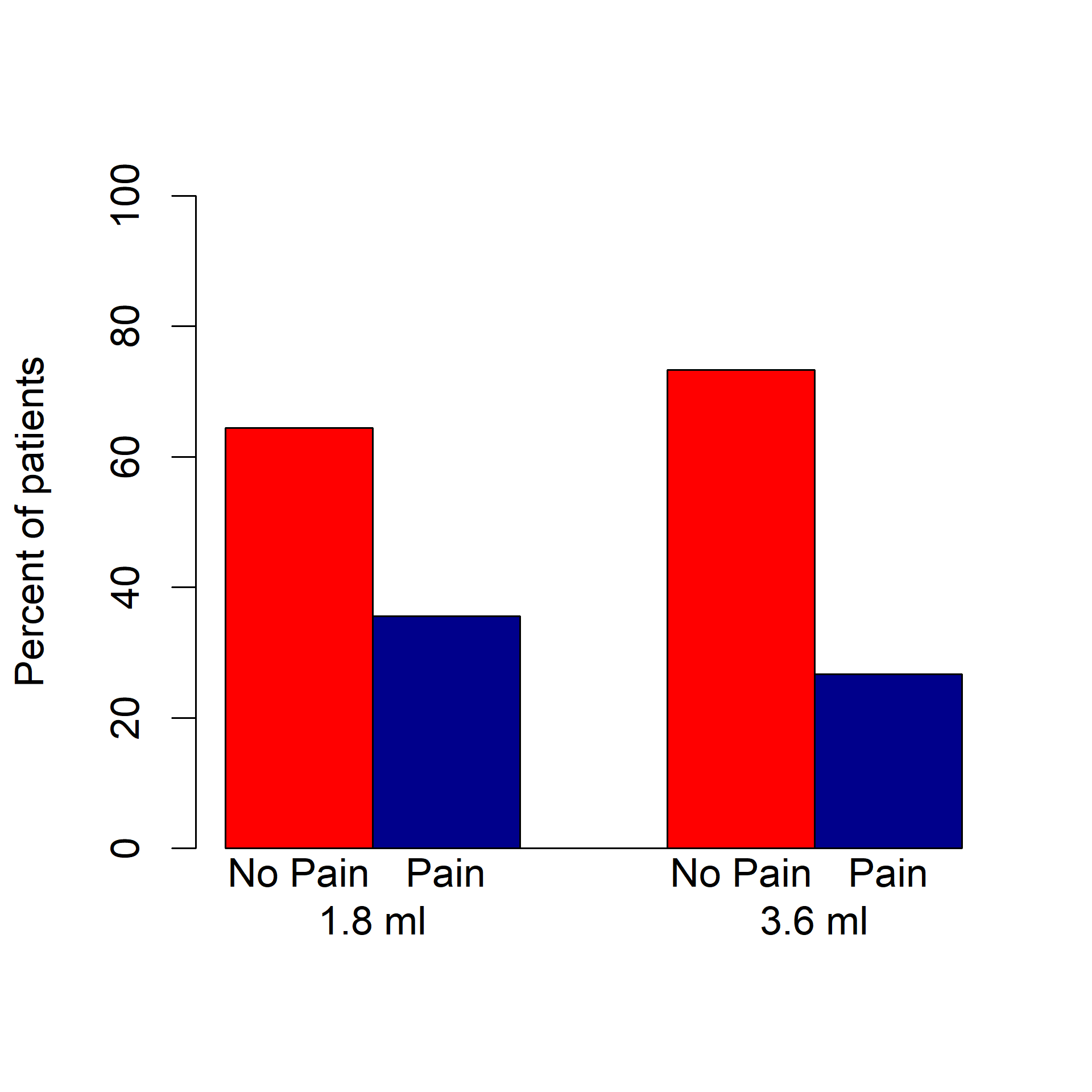

Supplement: S2 Table — (TIF) [file pone.0219536.s006.TIF]

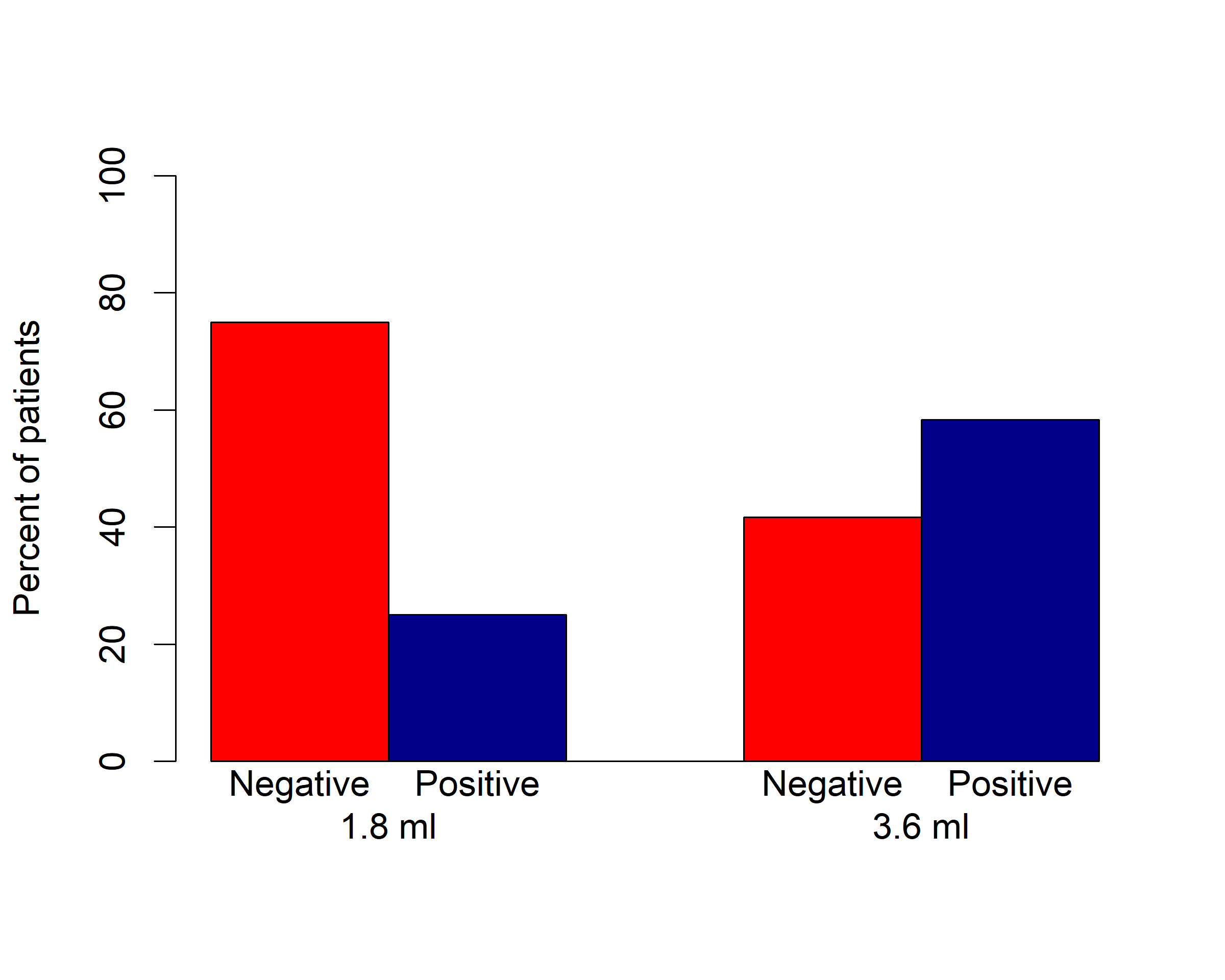

Supplement: S3 Table — (TIF) [file pone.0219536.s007.TIF]

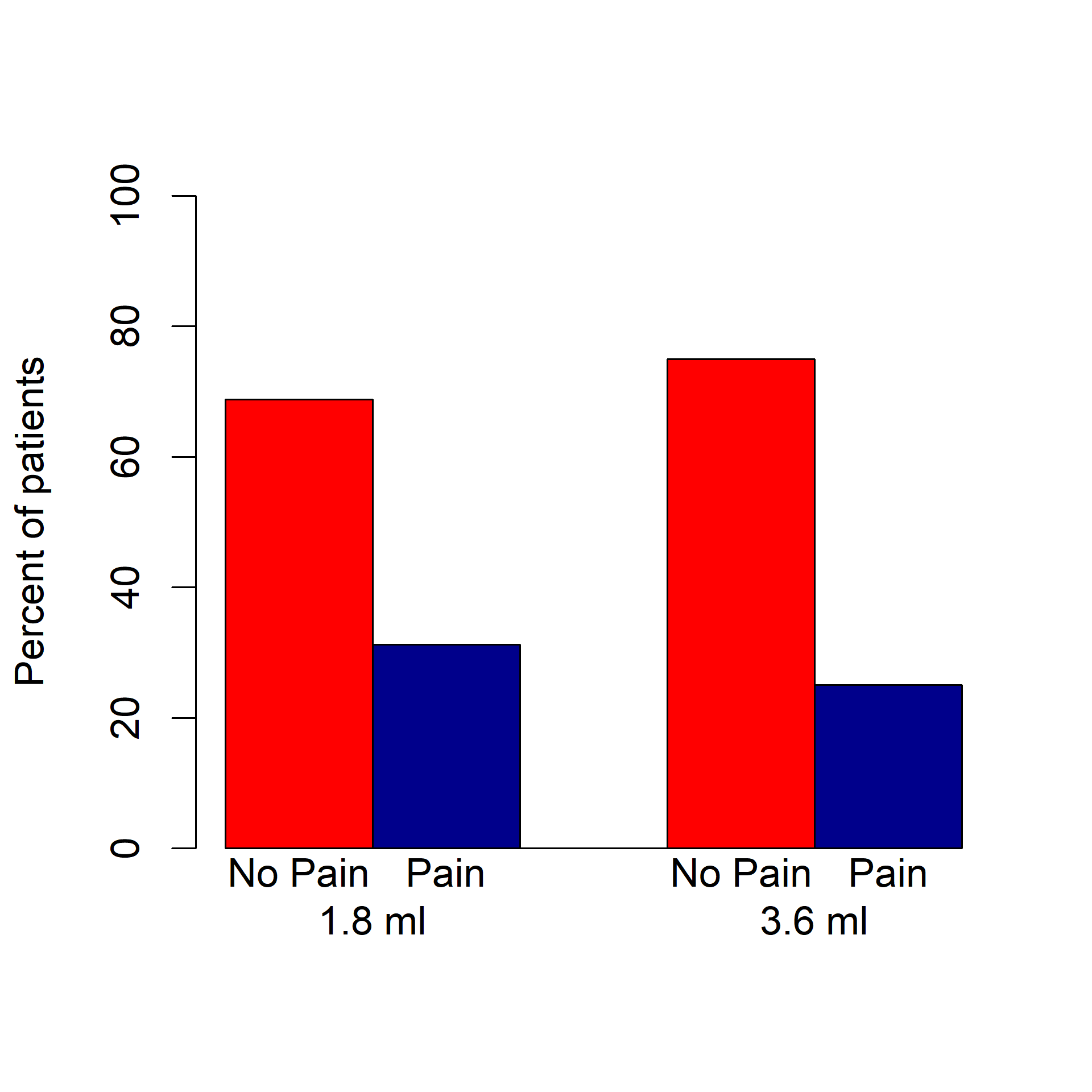

Supplement: S4 Table — (TIF) [file pone.0219536.s008.TIF]
