# Supplementary material for: Comparative evaluation of anesthetic efficacy of 1.8 mL and 3.6 mL of articaine in irreversible pulpitis of the mandibular molar: A randomized clinical trial
Source: PLoS One. 2019 Jul 31;14(7):e0219536. doi: 10.1371/journal.pone.0219536 (PMC6668778; doi:10.1371/journal.pone.0219536)
Supplement: S1 Protocol — (PDF) [file pone.0219536.s009.pdf]

ClinicalTrials.gov PRS
Protocol Registration and Results System

ClinicalTrials.gov PRS DRAFT Receipt (Working Version)
Last Update: 03/12/2019 13:00

ClinicalTrials.gov ID: NCT02422823

Study Identification

Unique Protocol ID: Volumes

Brief Title: Anesthetic Efficacy of 1,8mL and 3,6mL of Articaine in Inferior Alveolar Nerve Block in Irreversible Pulpitis (Volumes)

Official Title: Comparative Evaluation of the Anesthetic Efficacy of 1,8mL and 3,6mL of 4% Articaine With 1: 100,000 Epinephrine in Blocking the Inferior Alveolar Nerve in Patients With Irreversible Pulpitis of Mandibular Molars

Secondary IDs:

Study Status

Record Verification: March 2019

Overall Status: Completed

Study Start: April 2015 []

Primary Completion: June 2016 [Actual]

Study Completion: June 2016 [Actual]

Sponsor/Collaborators

Sponsor: Isabel Peixoto Tortamano

Responsible Party: Sponsor-Investigator
Investigator: Isabel Peixoto Tortamano [itortamano]
Official Title: Associate Professor
Affiliation: University of Sao Paulo

Collaborators:

Oversight

U.S. FDA-regulated Drug:

U.S. FDA-regulated Device:

U.S. FDA IND/IDE: No

Human Subjects Review: Board Status: Approved
Approval Number: 626.279
Board Name: Comitê de Etica em pesquisa da FOUSP
Board Affiliation: Universidade de Sao Paulo
Phone: 55-11-30917960
Email: cepfo@usp.br
Address:

Avenida Professor Lineu Prestes, 2227

Data Monitoring: No

FDA Regulated Intervention: No

Study Description

Brief Summary: Ninety patients with irreversible pulpitis diagnostic will participate in this clinical study. The participants will be divided into 2 groups of 45 patients, who will receive the inferior alveolar nerve block injections of 1.8 mL of 4% articaine (Articaine 100; DFL, Rio de Janeiro, RJ, Brazil) with 1:100,000 epinephrine or 3.6mL of the same solution. Two consecutive negative responses to the maximum pulp stimulus (80 µA) at the electric pulp test were the criterion to determine a pulpal anesthesia as successful. Ten minutes after the IAN block, subjective lip anesthesia will be evaluated by asking the patient whether his/her lip was numb. Thereafter and immediately before the pulpectomy, the electric pulp stimulations will be repeated to determine pulpal anesthesia. During the pulpectomy procedure, the patients were instructed to report any painful discomfort. To evaluate the intensity of pain during the pulpectomy, a verbal analogue scale will be used. The anesthesia will be defined as successful when the dentist accessed the pulp chamber without pain being reported by the patient. In these cases, the pulpectomy will be continued. If report pain will classified the IAN block as unsuccessful.

Detailed Description: Ninety patients will Participate in this clinical study. The patients will be admitted to the Emergency Center of the School of Dentistry at the University of São Paulo with a clinical diagnosis of irreversible pulpitis, The study was approved by the Committee on the Ethics of Research on Human Beings of the School of Dentistry at the University of São Paulo (protocol 95/07), and each patient will be informed to sing consent to participate in the study. The 90 participants will be divided into 2 groups of 45 patients, who will receive the inferior alveolar nerve block injections of 1.8 mL (equivalent to 1 cartridge) of 4% articaine (Articaine 100; DFL, Rio de Janeiro, RJ, Brazil) with 1:100,000 epinephrine or 3.6mL (equivalent to 2 cartridges) of the same solution. Two consecutive negative responses to the maximum pulp stimulus (80 µA) at the electric stimulation were the criterion to determine a pulpal anesthesia as successful. Before the IAN block injections, the tooth with irreversible pulpitis, the adjacent tooth, and the contralateral canine will be tested for pulp vitality with an electric pulp test (Vitality Scanner 2006; SybronEndo, Orange, CA). The electric pulp stimulation of the contralateral canine, which will be not anesthetized, will be used as control to ensure that the equipment is working properly and that patients is responding adequately.

The average injection time for each cartridge was approximately 2 minutes. Ten minutes after the IAN block, subjective lip anesthesia will be evaluated by asking the patient whether his/her lip was numb. Thereafter and immediately before the pulpectomy, the electric pulp stimulations will be repeated to determine pulpal anesthesia. During the pulpectomy procedure, the patients were instructed to report any painful discomfort. To evaluate the intensity of pain during the pulpectomy, a verbal analogue scale will be used: 0, no pain; 1, mild, bearable pain; 2, moderate, unbearable pain; 3, severe, intense, and unbearable pain. The anesthesia will be defined as successful when the dentist accessed the pulp chamber without pain being reported by the patient (pain scores 0 or 1). In these cases, the pulpectomy will be continued. Pain scores of 2 or 3 will classified the IAN block as unsuccessful.

Conditions

Conditions: Pulpitis

Keywords: anesthesia
volumes
articaine
Inferior alveolar nerve block

Study Design

Study Type: Interventional

Primary Purpose: Treatment

Study Phase: N/A

Interventional Study Model: Parallel Assignment

Number of Arms: 2

Masking: Double (Investigator, Outcomes Assessor)

Allocation: Randomized

Enrollment: 90 [Actual]

Arms and Interventions

Arms	Assigned Interventions
Experimental: Articaine 1.8mL<br>injections of 1.8 mL of 4% articaine with 1:100,000 epinephrine<br>Intervention: inferior alveolar nerve block injection	Drug: Articaine 1,8mL<br>Injection of 1.8mL of 4% articaine with 1: 100,000 epinephrine in block the inferior alveolar nerve in patients with irreversible pulpitis of mandibular molar in 45 patients<br>Other Names:<br>• Articaine 100, DFL, Rio de Janeiro, RJ, Brasil

Arms	Assigned Interventions
Active Comparator: Articaine 3.6mL<br>injections of 3.6 mL of 4% articaine with 1:100,000 epinephrine<br>Intervention: inferior alveolar nerve block injection	Drug: Articaine 3,6mL<br>Injection of 3.6mL of 4% articaine with 1: 100,000 epinephrine in block the inferior alveolar nerve in patients with irreversible pulpitis of mandibular molar in 45 patients<br>Other Names:<br>• Articaine 100, DFL, Rio de Janeiro, RJ, Brasil

Outcome Measures

[See Results Section.]

Eligibility

Minimum Age: 18 Years

Maximum Age: 50 Years

Sex: All

Gender Based:

Accepts Healthy Volunteers: Yes

Criteria: Inclusion Criteria:

- Patients 18-50 years, with mandibular molars with irreversible pulpitis, and have 1 molar adjacent to a molar presenting irreversible pulpites and a healthy contralateral canine with no deep carious lesions, extensive restoration, advanced periodontal disease, a history of trauma, or sensitivity.

Exclusion Criteria:

- Patients who took medication potentially interacting with the anesthetic used in the study

Contacts/Locations

Central Contact Person: Stella A Silva, DDS
Telephone: 11996462426
Email: stella.agra.silva@usp.br

Central Contact Backup:

Study Officials: Isabel P Tortamano
Study Principal Investigator
University of Sao Paulo

Locations:	**Brazil**<br>Faculdade de Odontologia da USP<br>Sao Paulo, SP, Brazil, 05508-000<br>Contact: Isabel P Tortamano, PHD 1130917813 iptortam@usp.br

IPDSharing

Plan to Share IPD:

References

Citations:

Links:

Available IPD/Information:

Study Results

Participant Flow

Recruitment Details	The pacients recuitment was between April 2015 to June 2016 in Emergency Center of the School of Dentistry at the University of Sao Paulo
Pre-assignment Details	All patients admitted to the Emergency Center of the School of Dentistry at the University of São Paulo, with a clinical diagnosis of irreversible pulpitis, which fulfilled eligibility criteria were included.

Reporting Groups

	Description
Injection of 1.8mL	injections of 1.8 mL of 4% articaine with 1:100,000 epinephrine Intervention: inferior alveolar nerve block injection<br>Articaine: Injection of 1.8mL of 4% articaine with 1: 100,000 epinephrine in block the inferior alveolar nerve in patients with irreversible pulpitis of mandibular molar in 40 patients<br>Epinephrine

	Description
Injection of 3.6mL	injections of 3.6 mL of 4% articaine with 1:100,000 epinephrine Intervention: inferior alveolar nerve block injection<br><br>Articaine: Injection of 3.6mL of 4% articaine with 1: 100,000 epinephrine in block the inferior alveolar nerve in patients with irreversible pulpitis of mandibular molar in 40 patients<br><br>Epinephrine

Overall Study

	Injection of 1.8mL	Injection of 3.6mL
Started	45	45
Completed [1]	45	45 [1]
Not Completed	0	0

[1] Time frame was 10 minutes after anesthesia, in the same session. So, there were no drop-outs

Baseline Characteristics

Reporting Groups

	Description
Injection of 1.8mL	injections of 1.8 mL of 4% articaine with 1:100,000 epinephrine Intervention: inferior alveolar nerve block injection<br><br>Articaine: Injection of 1.8mL of 4% articaine with 1: 100,000 epinephrine in block the inferior alveolar nerve in patients with irreversible pulpitis of mandibular molar in 40 patients<br><br>Epinephrine
Injection of 3.6mL	injections of 3.6 mL of 4% articaine with 1:100,000 epinephrine Intervention: inferior alveolar nerve block injection<br><br>Articaine: Injection of 3.6mL of 4% articaine with 1: 100,000 epinephrine in block the inferior alveolar nerve in patients with irreversible pulpitis of mandibular molar in 40 patients<br><br>Epinephrine

Baseline Measures

	Injection of 1.8mL	Injection of 3.6mL	Total
Overall Number of Participants	45	45	90

		Injection of 1.8mL	Injection of 3.6mL	Total
**Age, Categorical**<br>Measure Type: Count of Participants<br>Unit of measure: participants	Number Analyzed	45 participants	45 participants	90 participants
	<=18 years	0 0%	0 0%	0 0%
	Between 18 and 65 years	45 100%	45 100%	90 100%
	>=65 years	0 0%	0 0%	0 0%
**Sex: Female, Male**<br>Measure Type: Count of Participants<br>Unit of measure: participants	Number Analyzed	45 participants	45 participants	90 participants
	Female	27 60%	26 57.78%	53 58.89%
	Male	18 40%	19 42.22%	37 41.11%
**Race and Ethnicity Not Collected** [1]<br>Measure Type: Count of Participants<br>Unit of measure: participants	Number Analyzed	0 participants	0 participants	0 participants
		---	---	0
		[1] Measure Analysis Population Description: Race and Ethnicity were not collected from any participant.		
**Region of Enrollment**<br>Measure Type: Number<br>Unit of measure: participants	Number Analyzed	45 participants	45 participants	90 participants
Brazil		45	45	90

Outcome Measures

1. Primary Outcome Measure:

Measure Title	Patient Self-report of Pain

Measure Description	Subjects reported whether or not they had pain, using a verbal analogue scale, ranging from 0 to 3, where 3 is the worst pain.<br><br>0 and 1 was presented as "no pain". 2 and 3 was presented as "pain". Thus, the outcome is present as yes/no
Time Frame	intraoperative, measured 10 minutes after anesthesia

Analysis Population Description
[Not Specified]

Reporting Groups

	Description
Injection of 1.8mL	injections of 1.8 mL of 4% articaine with 1:100,000 epinephrine Intervention: inferior alveolar nerve block injection<br><br>Articaine: Injection of 1.8mL of 4% articaine with 1: 100,000 epinephrine in block the inferior alveolar nerve in patients with irreversible pulpitis of mandibular molar in 40 patients<br><br>Epinephrine
Injection of 3.6mL	injections of 3.6 mL of 4% articaine with 1:100,000 epinephrine Intervention: inferior alveolar nerve block injection<br><br>Articaine: Injection of 3.6mL of 4% articaine with 1: 100,000 epinephrine in block the inferior alveolar nerve in patients with irreversible pulpitis of mandibular molar in 40 patients<br><br>Epinephrine

Measured Values

	Injection of 1.8mL	Injection of 3.6mL
Overall Number of Participants Analyzed	45	45
Patient Self-report of Pain<br>Measure Type: Count of Participants<br>Unit of measure: participants	45 100%	45 100%

Statistical Analysis 1 for Patient Self-report of Pain

Statistical Analysis Overview	Comparison Group Selection	Injection of 1.8mL, Injection of 3.6mL
	Comments	[Not specified]
	Type of Statistical Test	Superiority
	Comments	Comparison of the number of subjects in each group that reported pain 10 min after anesthesia, by means of chi-square test

Statistical Test of Hypothesis	P-Value	<0.05
	Comments	[Not specified]
	Method	Chi-squared
	Comments	[Not specified]

Method of Estimation	Estimation Parameter	Odds Ratio (OR)
	Estimated Value	2.0096
	Confidence Interval	(2-Sided) 95%<br>0.8279 to 4.8782
	Estimation Comments	[Not specified]

Reported Adverse Events

Time Frame	End of procedure (same session)
Adverse Event Reporting Description	[Not specified]

Reporting Groups

	Description
Injection of 1.8mL	injections of 1.8 mL of 4% articaine with 1:100,000 epinephrine Intervention: inferior alveolar nerve block injection<br><br>Articaine: Injection of 1.8mL of 4% articaine with 1: 100,000 epinephrine in block the inferior alveolar nerve in patients with irreversible pulpitis of mandibular molar in 40 patients<br><br>Epinephrine
Injection of 3.6mL	injections of 3.6 mL of 4% articaine with 1:100,000 epinephrine Intervention: inferior alveolar nerve block injection<br><br>Articaine: Injection of 3.6mL of 4% articaine with 1: 100,000 epinephrine in block the inferior alveolar nerve in patients with irreversible pulpitis of mandibular molar in 40 patients<br><br>Epinephrine

All-Cause Mortality

	Injection of 1.8mL	Injection of 3.6mL
	Affected/At Risk (%)	Affected/At Risk (%)
Total All-Cause Mortality	0/45 (0%)	0/45 (0%)

Serious Adverse Events

	Injection of 1.8mL	Injection of 3.6mL
	Affected/At Risk (%)	Affected/At Risk (%)
Total	0/45 (0%)	0/45 (0%)

Other Adverse Events

Frequency Threshold Above Which Other Adverse Events are Reported: 0%

	Injection of 1.8mL	Injection of 3.6mL
	Affected/At Risk (%)	Affected/At Risk (%)
Total	0/45 (0%)	0/45 (0%)

Limitations and Caveats

[Not specified]

More Information

Certain Agreements:

All Principal Investigators ARE employed by the organization sponsoring the study.

Results Point of Contact:

Name/Official Title: Dr. Isabel Peixoto Tortamano
Organization: USaoPaulo
Phone: 55 11 30917418
Email: iptortam@usp.br
